# Genome sequence of *Ensifer medicae* strain WSM1369; an effective microsymbiont of the annual legume *Medicago sphaerocarpos*

**DOI:** 10.4056/sigs.4838624

**Published:** 2013-12-17

**Authors:** Jason Terpolilli, Giovanni Garau, Yvette Hill, Rui Tian, John Howieson, Lambert Bräu, Lynne Goodwin, James Han, Konstantinos Liolios, Marcel Huntemann, Amrita Pati, Tanja Woyke, Konstantinos Mavromatis, Victor Markowitz, Natalia Ivanova, Nikos Kyrpides, Wayne Reeve

**Affiliations:** 1 Centre for Rhizobium Studies, Murdoch University, Western Australia, Australia; 2 Dipartimento di Agraria, S.T.A.A., University of Sassari, Italy; 3 School of Life and Environmental Sciences, Deakin University, Victoria, Australia; 4 Los Alamos National Laboratory, Bioscience Division, Los Alamos, New Mexico, USA; 5 DOE Joint Genome Institute, Walnut Creek, California, USA; 6 Biological Data Management and Technology Center, Lawrence Berkeley National Laboratory, Berkeley, California, USA

**Keywords:** root-nodule bacteria, nitrogen fixation, rhizobia, *Alphaproteobacteria*

## Abstract

*Ensifer medicae* WSM1369 is an aerobic, motile, Gram-negative, non-spore-forming rod that can exist as a soil saprophyte or as a legume microsymbiont of *Medicago*. WSM1369 was isolated in 1993 from a nodule recovered from the roots of *Medicago sphaerocarpos* growing at San Pietro di Rudas, near Aggius in Sardinia (Italy). WSM1369 is an effective microsymbiont of the annual forage legumes *M. polymorpha* and *M. sphaerocarpos*. Here we describe the features of *E. medicae* WSM1369, together with genome sequence information and its annotation. The 6,402,557 bp standard draft genome is arranged into 307 scaffolds of 307 contigs containing 6,656 protein-coding genes and 79 RNA-only encoding genes. This rhizobial genome is one of 100 sequenced as part of the DOE Joint Genome Institute 2010 Genomic Encyclopedia for Bacteria and Archaea-Root Nodule Bacteria (GEBA-RNB) project.

## Introduction

One of the key nutritional constraints to plant growth and development is the availability of nitrogen (N) in nutrient deprived soils [1]. Although the atmosphere consists of approximately 80% N, the overwhelming proportion of this is present in the form of dinitrogen (N_2_) which is biologically inaccessible to most plants and other higher organisms. Before the development of the Haber-Bosch process, the primary mechanism for converting atmospheric N_2_ into a bioaccessible form was via biological nitrogen fixation (BNF) [2]. In BNF, N_2_ is made available by specialized microbes that possess the necessary molecular machinery to reduce N_2_ into NH_3_. Some plants, most of which are legumes, have harnessed BNF by evolving symbiotic relationships with specific N_2_-fixing microbes (termed rhizobia) whereby the host plant houses the bacteria in root nodules, supplying the microsymbiont with carbon and in return receives essential reduced N-containing products [3]. When BNF is exploited in agriculture, some of this N_2_ fixed into plant tissues is ultimately released into the soil following harvest or senescence, where it can then be assimilated by subsequent crops. Compared to industrially synthesized N-based fertilizers, BNF is a low energy, low cost and low greenhouse-gas producing alternative and hence its application is crucial to increasing the environmental and economic sustainability of farming systems [4].

Forage and fodder legumes play vital roles in sustainable farming practice, with approximately 110 million ha under production worldwide [5], a significant proportion of which is made up by members of the genus *Medicago*. *Ensifer meliloti* and *E. medicae* are known to nodulate and fix N_2_ with *Medicago* spp [6], although they have differences in host specificity. While *E. meliloti* strains do not nodulate *M. murex*, nodulate but do not fix N_2_ with *M. polymorpha* and nodulate but fix very poorly with *M. arabica* [7,8], they are able to nodulate and fix N_2_ with *Medicago* species originating from alkaline soils including the perennial *M. sativa* and the annuals *M. littoralis* and *M*. *tornata* [9,10]. In contrast, *E. medicae* strains can nodulate and fix N_2_ with annuals well adapted to acidic soils, such as *M. murex, M. arabica and M. polymorpha* [7,8].

The *E. medicae* strain WSM1369 was isolated from a nodule collected from *M. sphaerocarpos* growing at San Pietro di Rudas, near Aggius in Sardinia (Italy). This strain nodulates and fixes N_2_ effectively with *M. polymorpha* and *M. sphaerocarpos* [8]. Like *M. murex* and *M. polymorpha*, *M. sphaerocarpos* is an annual species which is tolerant of low pH soils [11], with studies suggesting that it only establishes N_2_-fixing associations with *E. medicae* strains [8,9]. However, owing to a paucity of symbiotic information, it is not yet clear whether *M. sphaerocarpos* fixes N_2_ with a wide range of *E. medicae* strains or if this ability is restricted to a smaller set of *E. medicae* accessions. Therefore, genome sequences of *E. medicae* strains effective with *M. sphaerocarpos* will provide a valuable genetic resource to further investigate the symbiotaxonomy of *Medicago-*nodulating rhizobia and will further enhance the existing available genome data for *Ensifer* microsymbionts [12-15]. Here we present a summary classification and a set of general features for this microsymbiont together with a description of its genome sequence and annotation.

## Classification and features

**Figure 1:**
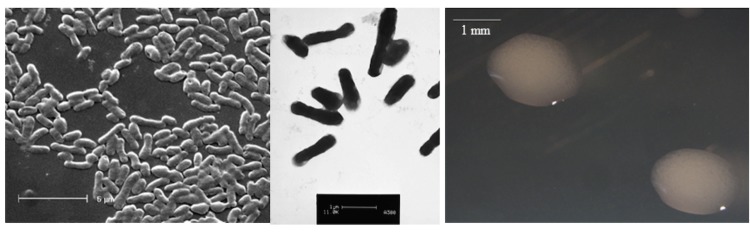
Images of *Ensifer medicae* WSM1369 using scanning (Left) and transmission (Center) electron microscopy and the appearance of colony morphology on half strength lupin agar (Right).

*E. medicae* WSM1369 is a motile, non-sporulating, non-encapsulated, Gram-negative rod in the order *Rhizobiales* of the class *Alphaproteobacteria*. The rod-shaped form varies in size with dimensions of approximately 0.25-0.5 μm in width and 1.0-1.5 μm in length (Figure 1 Left and 1 Center). It is fast growing, forming colonies within 3-4 days when grown on TY agar [16] or half strength Lupin Agar (½LA) [17] at 28°C. Colonies on ½LA are opaque, slightly domed and moderately mucoid with smooth margins (Figure 1 Right).

**Figure 2:**
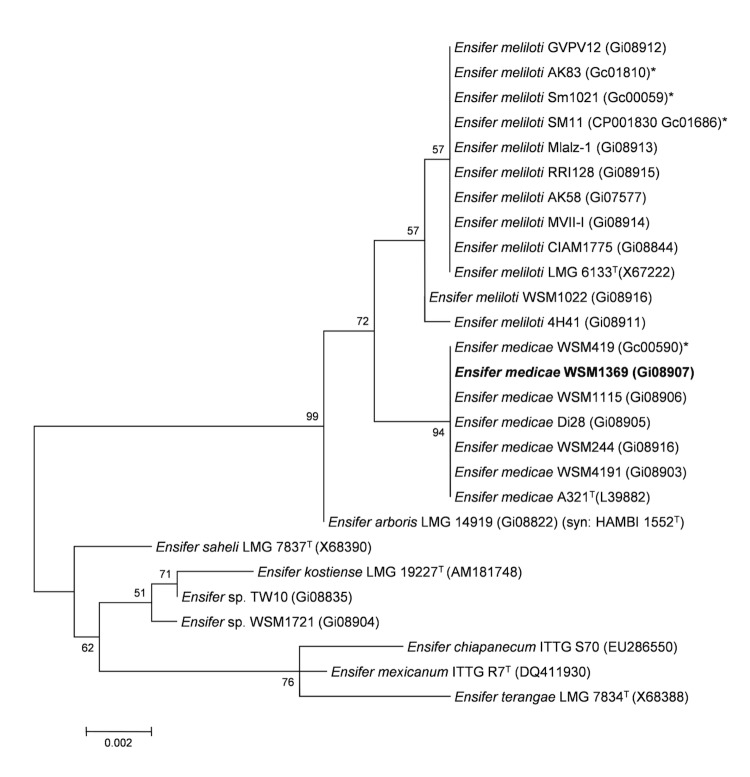
Phylogenetic tree showing the relationship of *Ensifer medicae* WSM1369 (shown in bold print) to other *Ensifer* spp. in the order *Rhizobiales* based on aligned sequences of the 16S rRNA gene (1,290 bp internal region). All sites were informative and there were no gap-containing sites. Phylogenetic analyses were performed using MEGA, version 5 [31]. The tree was built using the Maximum-Likelihood method with the General Time Reversible model [32]. Bootstrap analysis [33] with 500 replicates was performed to assess the support of the clusters. Type strains are indicated with a superscript T. Brackets after the strain name contain a DNA database accession number and/or a GOLD ID (beginning with the prefix G) for a sequencing project registered in GOLD [34]. Published genomes are indicated with an asterisk.

Minimum Information about the Genome Sequence (MIGS) is provided in Table 1. Figure 2 shows the phylogenetic neighborhood of *E. medicae* WSM1369 in a 16S rRNA sequence based tree. This strain shares 100% sequence identity (over 1290 bp) to the 16S rRNA of *E. medicae* A321^T^ and *E. medicae* WSM419 [13] and 99% sequence identity (1362/1366 bp) to the 16S rRNA of *E. meliloti* Sm1021 [12].

**Table 1:** Classification and general features of *Ensifer medicae* WSM1369 according to the MIGS recommendations [18]

**MIGS ID**	**Property**	**Term**	**Evidence code**
	Current classification	Domain *Bacteria*	TAS [19]
		Phylum *Proteobacteria*	TAS [20]
		Class *Alphaproteobacteria*	TAS [21,22]
		Order *Rhizobiales*	TAS [21,23]
		Family *Rhizobiaceae*	TAS [24,25]
		Genus *Ensifer*	TAS [26-28]
		Species *Ensifer medicae*	TAS [27]
		Strain WSM1369	TAS [8]
	Gram stain	Negative	IDA
	Cell shape	Rod	IDA
	Motility	Motile	IDA
	Sporulation	Non-sporulating	NAS
	Temperature range	Mesophile	NAS
	Optimum temperature	28°C	IDA
	Salinity	Non-halophile	NAS
MIGS-22	Oxygen requirement	Aerobic	TAS [8]
	Carbon source	Varied	NAS
	Energy source	Chemoorganotroph	NAS
MIGS-6	Habitat	Soil, root nodule, on host	NAS
MIGS-15	Biotic relationship	Free living, symbiotic	TAS [8]
MIGS-14	Pathogenicity	Non-pathogenic	NAS
	Biosafety level	1	TAS [29]
	Isolation	Root nodule	TAS [8]
MIGS-4	Geographic location	Sardinia, Italy	TAS [8]
MIGS-5	Soil collection date	28 April 1993	IDA
MIGS-4.1 MIGS-4.2	Longitude Latitude	9.019167 40.971667	IDA IDA
MIGS-4.3	Depth	0-10 cm	IDA
MIGS-4.4	Altitude	Not recorded	IDA

Evidence codes – IDA: Inferred from Direct Assay; TAS: Traceable Author Statement (i.e., a direct report exists in the literature); NAS: Non-traceable Author Statement (i.e., not directly observed for the living, isolated sample, but based on a generally accepted property for the species, or anecdotal evidence). These evidence codes are from the Gene Ontology project [30].

### Symbiotaxonomy

*E. medicae* strain WSM1369 was isolated in 1993 from a nodule collected from the annual *M. sphaerocarpos* growing at San Pietro di Rudas, near Aggius, Sardinia in Italy (J. G. Howieson, pers. comm.). The site of collection was undulating grassland, with a soil derived from granite materials that had a depth of 20-40 cm and a pH of 6.0. The soil was a loamy-sand and *Lathyrus* and *Trifolium* spp. grew in association with *M. sphaerocarpos.* WSM1369 forms nodules (Nod^+^) and fixes N_2_ (Fix^+^) with *M. polymorpha* and *M. sphaerocarpos* [8].

## Genome sequencing and annotation

### Genome project history

This organism was selected for sequencing on the basis of its environmental and agricultural relevance to issues in global carbon cycling, alternative energy production, and biogeochemical importance, and is part of the Community Sequencing Program at the U.S. Department of Energy, Joint Genome Institute (JGI) for projects of relevance to agency missions. The genome project is deposited in the Genomes OnLine Database [34] and a standard draft genome sequence in IMG. Sequencing, finishing and annotation were performed by the JGI. A summary of the project information is shown in Table 2.

**Table 2:** Genome sequencing project information for *E. medicae* WSM1369

**MIGS ID**	**Property**	**Term**
MIGS-31	Finishing quality	Standard draft
MIGS-28	Libraries used	One Illumina fragment library
MIGS-29	Sequencing platforms	Illumina HiSeq 2000
MIGS-31.2	Sequencing coverage	Illumina: 321×
MIGS-30	Assemblers	Velvet version 1.1.04; Allpaths-LG version r39750
MIGS-32	Gene calling methods	Prodigal 1.4
	GenBank	AQUS00000000
	GenBank release date	August 28, 2013
	GOLD ID	Gi08907
	NCBI project ID	165337
	Database: IMG	2513237156
	Project relevance	Symbiotic N_2_ fixation, agriculture

### Growth conditions and DNA isolation

*E. medicae* WSM1369 was cultured to mid logarithmic phase in 60 ml of TY rich medium on a gyratory shaker at 28°C [35]. DNA was isolated from the cells using a CTAB (Cetyl trimethyl ammonium bromide) bacterial genomic DNA isolation method [36].

### Genome sequencing and assembly

The genome of *Ensifer medicae* WSM1369 was sequenced at the Joint Genome Institute (JGI) using Illumina technology [37]. An Illumina standard shotgun library was constructed and sequenced using the Illumina HiSeq 2000 platform which generated 13,712,318 reads totaling 2,057 Mbp.

All general aspects of library construction and sequencing performed at the JGI can be found at the JGI user home [36]. All raw Illumina sequence data was passed through DUK, a filtering program developed at JGI, which removes known Illumina sequencing and library preparation artifacts (Mingkun, L., Copeland, A. and Han, J., unpublished). The following steps were then performed for assembly: (1) filtered Illumina reads were assembled using Velvet [38] (version 1.1.04), (2) 1–3 Kbp simulated paired end reads were created from Velvet contigs using wgsim [39], (3) Illumina reads were assembled with simulated read pairs using Allpaths–LG [40] (version r39750). Parameters for assembly steps were: 1) Velvet (velveth: 63 –shortPaired and velvetg: –veryclean yes –exportFiltered yes –mincontiglgth 500 –scaffolding no–covcutoff 10) 2) wgsim (-e 0 -1 76 -2 76 -r 0 -R 0 -X 0) 3) Allpaths–LG (PrepareAllpathsInputs:PHRED64=1 PLOIDY=1 FRAGCOVERAGE=125 JUMPCOVERAGE=25 LONGJUMPCOV=50, RunAllpath-sLG: THREADS=8 RUN=stdshredpairs TARGETS=standard VAPIWARNONLY=True OVERWRITE=True). The final draft assembly contained 307 contigs in 307 scaffolds. The total size of the genome is 6.4 Mbp and the final assembly is based on 2,057 Mbp of Illumina data, which provides an average 321× coverage of the genome.

### Genome annotation

Genes were identified using Prodigal [41] as part of the DOE-JGI annotation pipeline [42]. The predicted CDSs were translated and used to search the National Center for Biotechnology Information (NCBI) nonredundant database, UniProt, TIGRFam, Pfam, PRIAM, KEGG, COG, and InterPro databases. The tRNAScanSE tool [43] was used to find tRNA genes, whereas ribosomal RNA genes were found by searches against models of the ribosomal RNA genes built from SILVA [44]. Other non–coding RNAs such as the RNA components of the protein secretion complex and the RNase P were identified by searching the genome for the corresponding Rfam profiles using INFERNAL [45]. Additional gene prediction analysis and manual functional annotation was performed within the Integrated Microbial Genomes (IMG-ER) platform [46].

## Genome properties

**Figure 3:**
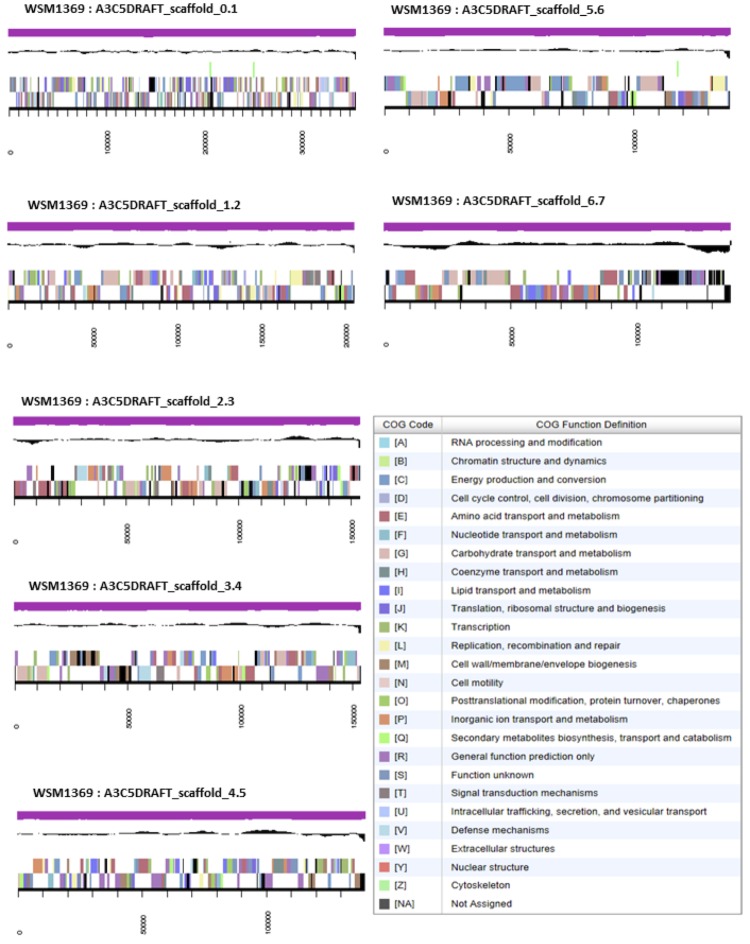
Graphical map of the genome of *Ensifer medicae* WSM1369 showing the seven largest scaffolds. From bottom to the top of each scaffold: Genes on forward strand (color by COG categories as denoted by the IMG platform), Genes on reverse strand (color by COG categories), RNA genes (tRNAs green, sRNAs red, other RNAs black), GC content, GC skew.

The genome is 6,402,557 nucleotides with 61.13% GC content (Table 3) and comprised of 307 scaffolds (Figure 3) of 307 contigs. From a total of 6,735 genes, 6,656 were protein encoding and 79 RNA only encoding genes. The majority of genes (74.14%) were assigned a putative function while the remaining genes were annotated as hypothetical. The distribution of genes into COGs functional categories is presented in Table 4.

**Table 3:** Genome Statistics for *Ensifer medicae* WSM1369

**Attribute**	**Value**	**% of Total**
Genome size (bp)	6,402,557	100.00
DNA coding region (bp)	5,536,774	86.48
DNA G+C content (bp)	3,913,921	61.13
Number of scaffolds	307	
Number of contigs	307	
Total gene	6,735	100.00
RNA genes	79	1.17
rRNA operons	1	0.01
Protein-coding genes	6,656	98.83
Genes with function prediction	4,993	74.14
Genes assigned to COGs	4,988	74.06
Genes assigned Pfam domains	5,185	76.99
Genes with signal peptides	508	7.54
Genes coding transmembrane proteins	1,424	21.14
CRISPR repeats	0	

**Table 4:** Number of protein coding genes of *Ensifer medicae* WSM1369 associated with the general COG functional categories.

**Code**	Value	**% age**	**Description**
J	193	3.48	Translation, ribosomal structure and biogenesis
A	0	0.00	RNA processing and modification
K	486	8.77	Transcription
L	275	4.96	Replication, recombination and repair
B	1	0.02	Chromatin structure and dynamics
D	40	0.72	Cell cycle control, mitosis and meiosis
Y	0	0.00	Nuclear structure
V	54	0.97	Defense mechanisms
T	241	4.35	Signal transduction mechanisms
M	267	4.82	Cell wall/membrane biogenesis
N	77	1.39	Cell motility
Z	0	0.00	Cytoskeleton
W	1	0.02	Extracellular structures
U	124	2.24	Intracellular trafficking and secretion
O	184	3.32	Posttranslational modification, protein turnover, chaperones
C	308	5.56	Energy production conversion
G	510	9.21	Carbohydrate transport and metabolism
E	613	11.06	Amino acid transport metabolism
F	108	1.95	Nucleotide transport and metabolism
H	196	3.54	Coenzyme transport and metabolism
I	193	3.48	Lipid transport and metabolism
P	280	5.05	Inorganic ion transport and metabolism
Q	158	2.85	Secondary metabolite biosynthesis, transport and catabolism
R	662	11.95	General function prediction only
S	569	10.27	Function unknown
-	1,747	25.94	Not in COGS
